# Could Brain–Computer Interface Be a New Therapeutic Approach for Body Integrity Dysphoria?

**DOI:** 10.3389/fnhum.2021.699830

**Published:** 2021-08-11

**Authors:** Stuti Chakraborty, Gianluca Saetta, Colin Simon, Bigna Lenggenhager, Kathy Ruddy

**Affiliations:** ^1^Occupational Therapy, Department of Physical Medicine and Rehabilitation, Christian Medical College and Hospital, Vellore, India; ^2^Department of Psychology, University of Zurich, Zurich, Switzerland; ^3^Trinity College Institute of Neuroscience and School of Psychology, Trinity College Dublin, Dublin, Ireland

**Keywords:** apotemnophilia, body integrity dysphoria, body integrity identity disorder, body representation, brain–computer interface, neurofeedback, somatoparaphrenia, xenomelia

## Abstract

Patients suffering from body integrity dysphoria (BID) desire to become disabled, arising from a mismatch between the desired body and the physical body. We focus here on the most common variant, characterized by the desire for amputation of a healthy limb. In most reported cases, amputation of the rejected limb entirely alleviates the distress of the condition and engenders substantial improvement in quality of life. Since BID can lead to life-long suffering, it is essential to identify an effective form of treatment that causes the least amount of alteration to the person’s anatomical structure and functionality. Treatment methods involving medications, psychotherapy, and vestibular stimulation have proven largely ineffective. In this hypothesis article, we briefly discuss the characteristics, etiology, and current treatment options available for BID before highlighting the need for new, theory driven approaches. Drawing on recent findings relating to functional and structural brain correlates of BID, we introduce the idea of brain–computer interface (BCI)/neurofeedback approaches to target altered patterns of brain activity, promote re-ownership of the limb, and/or attenuate stress and negativity associated with the altered body representation.

## Introduction

For most people, the thought of becoming physically disabled is troubling and something to be avoided at all costs. For patients suffering from body integrity dysphoria (BID) however, the desire to become disabled is characteristic, arising from a mismatch between the desired body and the physical body in terms of functionality or shape. In its most common variant, i.e., the desire for amputation, on which we focus here, the defining features include an intense feeling of inappropriateness concerning their current body configuration. BID patients desire to become physically disabled in order to feel “complete” and become the person they envisage themselves to be (First, 2005; White, 2014). Ironically, achieving this completeness often involves desiring to amputate an otherwise healthy and fully functioning limb, raising many ethical and medical considerations. In most reported cases, amputation of the rejected limb entirely alleviates the distress of the condition and engenders substantial improvement in quality of life (Noll and Kasten, 2014). Individuals with BID are non-psychotic and do not otherwise present major psychiatric or neurological disorders. Sufferers often describe themselves as being *trans-*abled, drawing a parallel with transgendered individuals.

Since BID can lead to life-long suffering, it is essential to identify an effective form of treatment that causes the least amount of alteration to the person’s anatomical structure and functionality. Current forms of treatment such as amputation are extreme and lead to a permanent disability and presumably dependence on carers for activities of daily living. Other treatment methods such as medications, psychotherapy, and vestibular stimulation have proven largely ineffective (Ryan, 2008; Blom et al., 2012). In this article, we briefly discuss the characteristics, etiology, and current treatment options available for BID before highlighting the need for new, theory driven approaches. Drawing on recent findings relating to functional and structural brain correlates of BID, we introduce the idea of brain–computer interface (BCI)/neurofeedback approaches to target altered patterns of brain activity, promote re-ownership of the limb, and/or attenuate stress and negativity associated with the altered body representation.

## Characteristics and Etiology of BID

In order to provide an in-depth evaluation of BID and its manifestations, it may be helpful to first differentiate from other similar conditions such as body dysmorphic disorder (BDD) and somatoparaphrenia, among others (Table 1). BID shares similarities with BDD, because individuals with BID are also found to be preoccupied with certain aspects of their body (Grocholewski et al., 2018). However, there are some key differences. The dysphoria experienced by individuals with BDD is derived from their concerns over how a part of their body physically appears to others (First and Fisher, 2012). Specifically, individuals with BDD believe that some aspect of their physical appearance is defective and as such is a source of intense embarrassment, shame, and low self-esteem.

**TABLE 1:** Taxonomy of disorders related to body integrity dysphoria.

**Disorder**	**Key features**		**Implicated brain regions**
Body integrity dysphoria (BID)	A “disorder of bodily distress or bodily experience” (World Health Organization, 2018). Desire to become physically disabled (e.g., blind, paralyzed, and amputated). Amputation variant includes (1) dysphoric feelings about a typical body morphology and/or functionality originating from a mismatch between the mental body image, i.e., the conscious representation of body size and shape, and the perceived body (Saetta et al., 2020), (2) lack of ownership over a limb, (3) excessive attention devoted to the limb, and (4) intact insight regarding the condition and its unusual nature (Giummarra et al., 2011). Most often left limb or both limbs (First, 2005). Typically observed in BID are also paraphilic interests, such as preferences for an amputated sexual partner and sexual arousal by the idea of being an amputee (Blom et al., 2017)		- Right superior parietal lobule (Ramachandran et al., 2009; McGeoch et al., 2011; Hilti et al., 2013; Saetta et al., 2020) - Left premotor ventral cortex (Saetta et al., 2020; van Dijk et al., 2013; Blom et al., 2016) - Right primary somatosensory and motor cortices (Hilti et al., 2013; Saetta et al., 2020) - Bilateral nucleus caudatus and putamen (Hänggi et al., 2016)
	Shared features with BID	Features distinct from BID	
Body dysmorphic disorder (BDD)	Preoccupation with certain aspects of the body (Grocholewski et al., 2018)	Dysphoria experienced by individuals with BDD is derived from their concerns over how a part of their body physically appears to others (First and Fisher, 2012). Specifically, individuals with BDD believe that some aspect of their physical appearance is defective and as such is a source of intense embarrassment, shame, and low self-esteem	- Reduced volume of the orbito-frontal cortex and the anterior cingulate, and larger volume of the thalamus (Atmaca et al., 2010) - Frontostriatal and temporoparietal occipital circuits for visual-spatial processing (Mufaddel et al., 2013)
Somatoparaphrenia	Denial of ownership over a limb (Gerstmann, 1942), and more often concerns the left body part (McGeoch et al., 2011).	Acquired condition in response to brain damage, typically right hemisphere, which may cause left limb paralysis. Characterized by delusional misattribution of the paralyzed limb to someone else (Gerstmann, 1942; Romano and Maravita, 2019)	- Right hemisphere subcortical white matter (Gandola et al., 2012; Moro et al., 2016) - Middle and inferior right frontal gyrus (Gandola et al., 2012) - Right hippocampus and amygdala (Gandola et al., 2012; Romano et al., 2014)
Asomatognosia	Disruption to right hemisphere influencing limb awareness Phenomenological similarities in 23% of the cases (Blanke and Metzinger, 2009)	Feeling that parts of the body are missing or have disappeared from corporal awareness (Arzy et al., 2006; Saetta et al., 2021)	Right temporo-parietal lobe, including the right superior parietal lobule (Saetta et al., 2021)
Alien hand syndrome	Disownership of the limb No traceable underlying cause for the perceived “disembodiment” of the limb (Müller, 2009)	Motor control of the affected limb is disinhibited and moves non-volitionally	- Primary motor cortex, premotor cortex, precuneus, and right inferior frontal gyrus (Schaefer et al., 2010) - Supplementary motor area, anterior cingulate gyrus, medial prefrontal cortex, and anterior corpus callosum (Feinberg et al., 1992)
Anarchic hand syndrome	Disembodiment of the limb Potential underlying mechanism relating to white matter disconnection and brain lesions (Pacella et al., 2021)	Motor control of the affected limb is disinhibited and moves non-volitionally No disownership of the limb	- Right inferior parietal lobe (Della Sala, 2009; Jenkinson et al., 2015) - White matter disconnection of antero-posterior, insular, and interhemispheric networks (Pacella et al., 2021) - Anterior corpus callosum (Feinberg et al., 1992)
Gender incongruity	Conflict between aspects of the physical body (sex) and the desired body. Onset (early childhood or adolescence), discontent in the individual with their identity, perceived reduction of desire post-surgical intervention or through mimicking of desired identity Comorbidity of BID with GID has been described in 19% of the cases (First, 2005; Lawrence, 2010) Preserved rationality and distress disappears after surgical intervention (Garcia-Falgueras, 2014)	Focus upon gender rather than upon a limb Intensity of rejection of body parts. Intense hatred in Gender incongruity vs. indifference in BID (Ostgathe et al., 2014)	- Bilateral superior parietal lobule and the primary somatosensory cortex (Lin et al., 2014) - Right insula (Nawata et al., 2010)

Somatoparaphrenia (SPP) is characterized by a denial of ownership combined with confabulatory signs (Gerstmann, 1942). It affects predominantly the left side of the body (McGeoch et al., 2011). Two main clinical features separate it from BID. First, while BID likely develops in childhood and may be related to amputation-related experiences (Barrow and Oyebode, 2019), SPP is an acquired condition in response to brain damage, typically of the right hemisphere, that typically also causes contralateral left limb paralysis. Moreover, while BID individuals recognize the unwanted limb as part of their current but undesired body, SPP is a disownership disorder, primarily characterized by a positive component, reflecting a “distorted mental representation of reality” (Bottini et al., 2009, p. 589), that is the delusional misattribution of the paralyzed limb to someone else (Gerstmann, 1942; Romano et al., 2014; Saetta et al., 2020). Another related clinical condition is asomatognosia, which is caused by a similar (more often a right lateral) cerebral lesion as SPP. In this condition, however, the limb is felt as non-existing rather than disowned (Critchley, 1954; Saetta et al., 2021).

Other conditions that have been discussed in relation to BID due to common features are alien hand syndrome and anarchic hand syndrome. For patients with alien hand syndrome, the motor control of the affected limb is disinhibited, and the limb executes smooth but non-volitional reflexive movements of grasping and compulsive manipulation of tools in the environment, i.e., utilization behavior (Doody and Jankovic, 1992; Feinberg et al., 1992). Typical damaged brain areas include fronto-mesial and anterior cingulate areas as well as the anterior corpus callosum (Feinberg et al., 1992). Anarchic hand syndrome (AHS) is a related disorder, which ensues from a relatively circumscribed lesion of the anterior corpus callosum (Feinberg et al., 1992, but see Pacella et al., 2021 for a more recent account). In this syndrome, the individuals’ hands move outside of the volitional control as well (Della Sala, 2009; Jenkinson et al., 2015); however, intermanual conflicts are more typical than utilization behavior.

Gender incongruity/dysphoria (GD) is a condition in which there is a conflict between aspects of the physical body (sex) and the desired body, often leading to sex-reassignment through surgery and lifelong hormonal treatment. Before the release of the DSM-V, GD was referred to as “Gender Identity Disorder.” The GD diagnosis separates this condition from sexual dysfunctions while framing it in terms of incongruence between phenomenological and assigned gender, instead of “cross-gender” identification disorder. Analogously, while BID has been previously defined as “Body Integrity Identity disorder” (First, 2005), the BID nomenclature focuses on the incongruence between the functionality and/or morphology of the current and assigned body. Conducting a direct comparison between attitudes of those with BID and GD (Ostgathe et al., 2014) revealed that both share similar onset (early childhood or adolescence), discontent in the individual with their identity, perceived reduction of desire post-surgical intervention, or through mimicking of desired identity such as cross-dressing in Gender Dysphoria and pretending of disability by using wheelchairs or crutches in BID. Comorbidity of BID with GID has been described in 19% of the cases (First, 2005; Lawrence, 2010). The groups differed, however, in the intensity of their aversion toward the unwanted body parts, with Gender Dysphoric individuals expressing an intense rejection or hatred, whereas BID individuals exhibited an indifference toward the limb. Remarkably, in both BID and GID individuals, the examination of reality is preserved in that the discomfort sensations are “illusions, not delusions” (Garcia-Falgueras, 2014; p. 161), and do not typically persist after surgical intervention (Garcia-Falgueras, 2014).

Before being recently included in the release of the 11th Revision of the International Classification of Diseases (World Health Organization, 2018) as a “disorder of bodily distress or bodily experience,” BID has also been interchangeably referred to as xenomelia, apotemnophilia, and body-integrity identity disorder (BIID). While the lowest common denominator between these nomenclatures is the desire for amputation, each of them underlies a specific etiological hypothesis (Sedda and Bottini, 2014).

Apotemnophilia (from ancient Greek “love for amputation”) proposes psychological or psychiatric features related to sexual disturbances to be the centerpiece of the disorder, whereas Xenomelia (from ancient Greek “foreign limb”) put the disorder in the context of a neurological syndrome originating from alterations of the right parietal lobe (McGeoch et al., 2011). A lack of consensus regarding the nomenclature concerned with these conditions and their etiological basis is a topic of ongoing scientific debate.

Major characteristics observed in the amputation variant of BID include (1) dysphoric feelings about a normal body morphology and/or functionality originating from a mismatch between the mental body image, i.e., the conscious representation of body size and shape, and the perceived body (Saetta et al., 2020), (2) lack of ownership over a limb, (3) excessive attention devoted to the limb, and (4) intact insight regarding the condition and its unusual nature (Giummarra et al., 2011). Initial studies on BID have shown that in the case of a desire for amputation, most patients with BID desired amputation of either a left-sided limb or both limbs (First, 2005).

Ostgathe et al. (2014) found that justifications given by those with BID desiring an amputation included triggering childhood events, restoring a congruence between sentiment and body, feeling attracted by people with physical disabilities, a fascination for physically impaired people, as well as the perception of body impairment as a proper way of living. Typically observed in BID are also paraphilic interests, such as preferences for an amputated sexual partner and sexual arousal by the idea of being an amputee (Blom et al., 2017).

## Current Approaches to Treatment for BID

Individuals with BID find symptomatic relief by spending substantial amounts of time engaged in so-called pretending behaviors in which they simulate the desired disability (e.g., walking with one’s leg tied back, sitting in a wheelchair, using crutches, First, 2005). It is believed that the pretending behavior allows for the alignment of the perceived and desired body, reducing distress associated with the mismatch (Saetta et al., 2020).

While this provides some transient relief, the preoccupation with becoming disabled has harmful consequences. Time spent engaging in pretending behaviors interferes with the individual’s productivity, leisure activities, and social functioning. In the case of desired amputation, pretending behaviors such as daily binding up of the leg can also cause injury over extended periods of time. At the most extreme end of the spectrum, attempts to actually achieve the desired disability can have catastrophic effects on the health of the individual, potentially putting their life at risk.

The only truly satisfying solution for BID sufferers seems to be the amputation of the rejected limb (Noll and Kasten, 2014). In most countries, this course of action will not be considered by physicians (Bayne and Levy, 2005) and is strictly illegal, knowing that it renders the individual with a new and unnecessary lifelong physical disability. Thus, alternative approaches targeting the comorbid anxiety and depression symptoms to reduce distress have been the key focus of treatment to date (e.g., Ryan, 2008; Blom et al., 2012). Psychotherapy has been shown to reduce psychological distress, but concurrently increase the individual’s desire for amputation, most likely as a result of intense focus and discussion on the topic of their BID with the therapist (Kröger et al., 2014).

Reports of the effectiveness of pharmacological interventions are very limited, and sample sizes are small, often limited to single case reports. One such case report suggested a reduction of distress in one individual with BID from a course of selective serotonin reuptake inhibitors (SSRIs), and limited effects of psychotherapy (Braam et al., 2006). Another report with 25 BID patients found that 12 had been prescribed medications including antidepressants, neuroleptic drugs, and tranquilizers but with no positive effects on symptoms (Kröger et al., 2014).

Another potential treatment that received prior attention was artificial vestibular stimulation, initially proposed by Ramachandran et al. (2007). As disorders of the bodily self have been linked to the vestibular system for over a century (Bonnier, 2009), artificial vestibular stimulation (caloric or galvanic) has been shown to induce temporary remission of the denial of ownership over a limb in SPP (Lopez, 2013) and other bodily self disorders (Grabherr et al., 2015). Given the above-mentioned parallels between the BID and SPP clinical pictures, vestibular stimulation has been tentatively used to restore limb ownership in BID and thus to reduce the desire for amputation. Despite initial excitement and early adoption of the technique clinically, subsequent research studies have demonstrated that it provides no substantial benefit to individuals with BID (Lenggenhager et al., 2014).

Upon review of the available literature, it is evident that aside from amputation, existing treatment options are largely ineffective, and fresh approaches are required to alleviate distress for those with BID. Furthermore, persisting with the current ineffective treatment programs comes at a financial cost to the patients and healthcare providers. Amputation, although effective, carries the largest financial burden at an estimated $24,010 for the procedure itself with inpatient stay, and $878,927 in associated costs over the lifetime of the amputated individual (United States data according to health insurance claims, Lo et al., 2020). With advances in neuroimaging, the field of BID is set to undergo a paradigm shift, availing of new knowledge on brain structural and functional correlates of the disorder that may provide insights toward potential avenues for treatment exploration. In the next sections, we review recent developments for understanding the neurological mechanisms and consider how these may inform the inception of new theory driven approaches to treatment.

## Structural and Functional Connectivity Abnormalities in BID

It has been well established since the mid 20th century that brain lesions in the right parietal lobe lead to altered perception and spatial awareness of body parts (Brain, 1941; Critchley, 1954). This area of the cortex is divided into the superior and inferior parietal lobules (the SPL and the IPL, respectively), with the latter being further subdivided into the supramarginal and angular gyri. Within the last decade, research specifically relating to structural and functional brain abnormalities in BID has emerged. In a recent review of all available neuroimaging evidence since 2012, Fornaro et al. (2020) reported that areas consistently found to be altered in xenomelia include right superior and inferior parietal lobule (rSPL and rIPL), premotor cortex, and right insula. A general pattern across studies emerged that cortical abnormalities tended to be right lateralized, whereas subcortical were left lateralized. Hänggi et al. (2016) specifically focused upon subcortical correlates of BID and found distinctive differences in the shape of several structures involved in somatotopic representation of the leg. Patients had thinner bilateral dorsomedial putamina, left ventromedial caudate nucleus, and left medial pallidum. This was accompanied by thicker bilateral lateral pallida and left frontolateral thalamus.

The aforementioned structural evidence is corroborated by data concerning functional connectivity between brain regions at rest in patients with BID compared to controls. In a relatively large and homogenous sample of BID patients who all desired amputation of the left leg, Saetta et al. (2020) found reduced functional connectivity between the primary sensorimotor area (S1) of the disowned leg and the rest of the brain, and the rSPL with the rest of the brain. Further, gray matter atrophy was evident in the rSPL of BID patients, and the extent of this atrophy correlated with desire for amputation and degree of engagement in pretending behaviors. These findings led Saetta et al. (2020) to suggest that the distressing feeling of non-acceptance of one’s limb in BID might ensue from a discrepancy between preserved projections of somatosensory inputs from the limb to the respective primary cortical areas, and an impaired representation of the body at the highest level of integration, the so-called body image. The authors proposed that only a smooth interplay between sensory and higher level bodily processing may provide the phenomenal experience of a limb belonging to the body and the self. The previously identified neural network for (healthy) body image and ownership has been suggested to span a broad network involving primary sensorimotor, premotor, parietal, insular, and extrastriatal areas (see, e.g., Tsakiris, 2010; Grivaz et al., 2017; Park and Blanke, 2019; Seghezzi et al., 2019; Salvato et al., 2020; for reviews), which were, at least to some extent, also found to be altered in BID (Fornaro et al., 2020).

The presence of underlying neuroanatomical structural abnormalities is evidence that BID has both psychological and neurophysiological components, and that recovery may entail modifying hard wired brain circuitry. However, this does not necessarily imply permanency of the condition as both gray and white matter are highly neuroplastic and structural changes over time can be documented following treatment programs for other neuropsychological disorders including depression (Liu et al., 2015; Bouckaert et al., 2016) and alcohol addiction (Pfefferbaum et al., 2014). By altering functional connectivity using targeted therapeutic interventions to promote desired neural patterns, changes in brain structure often follow over time. For example, using brain–computer interfaces, functional and structural neuroplastic changes can be induced in regions specifically involved in the trained task (Ghaziri et al., 2013; Qian et al., 2018; Marins et al., 2019; Nierhaus et al., 2021; Sampaio-Baptista et al., 2020).

## A New Generation of Research Into BID Treatment Using Neurofeedback/Brain–Computer Interface

Brain–computer interfaces and neurofeedback have been used in attempts to tune pathological brain rhythms or patterns of functional connectivity toward more neurotypical patterns (Ros et al., 2014). This is achieved by training individuals to use on-screen (or robotic) feedback of real-time brain activity to self-regulate some aspect of their own neural functioning. While BCI can come in a vast array of different arrangements using different neuroimaging modalities (see Simon et al., 2021 for a review), a commonly used setup involves recording electrical activity from the scalp using electroencephalography (EEG), and presenting feedback on screen in the form of a computer game. With this type of BCI, different aspects of brain activity can be targeted for modification depending on the method used to extract and present feedback. Positive outcomes using this method for symptom reduction have been demonstrated for ADHD (Qian et al., 2018), post-traumatic stress disorder (PTSD, van der Kolk et al., 2016; Banerjee and Argáez, 2017), and generalized anxiety disorder (GAD, Banerjee and Argáez, 2017; Mennella et al., 2017). In the case of BID, given that distinct functional connectivity patterns are evident involving S1, rSPL, and the rest of the brain (Saetta et al., 2020), there may be value in testing whether targeting this pattern using BCI would result in reduction of distress associated with the desire for amputation. For example, training could provide on-screen feedback in a computer game where rewards are achieved contingent on the strength of connectivity between the aforementioned nodes. Over multiple sessions playing the BCI game, strengthening of connectivity could be hypothesized as participants develop or refine mental strategies, and compared to a control condition where pseudofeedback is delivered. Experimental work would be essential to determine effective mental imagery strategies to recommend for participants (or alternatively whether to allow strategy-free implicit learning), and how many sessions of this type of feedback may be required in order to achieve symptomatic relief.

As Saetta et al. (2020) found that gray matter atrophy in BID correlated with the extent of pretending behaviors (such as binding up the leg and simulating amputation), and desire for amputation, it is also necessary to consider whether brain-related changes are a root cause of the disorder or an effect of the resultant coping behaviors. This too has implications for designing interventions based on structural or functional brain connectivity, as the intervention may be off target if focus is upon an epiphenomenal mechanism arising from the pretending behavior. Even if this is the case, however, BCI approaches to train functional connectivity patterns may still hold value. The simulation of the amputated state may in itself alter brain connectivity patterns for body representations, which may in turn enhance desire for amputation. BCI could be a means to disrupt this loop and find an alternative (less dangerous) method for symptomatic relief that does not perpetuate the altered activity in the brain’s body representation regions.

An alternative but related approach would be to use BCI to implicitly promote re-ownership of the limb and engender more positive associations with it. In a study using a foot-specific variant of the rubber hand illusion, Lenggenhager et al. (2015) demonstrated that individuals with BID behaved similarly to healthy controls in that they could take ownership for a fake rubber foot. When stroked synchronously with their opposite foot, they reported sensations as if it were part of their own body. This implies that in the unwanted limb, not only are the basic senses functioning normally but also that the integration of visual, tactile, and proprioceptive information and visual capture is broadly intact. Thus, the authors suggested that behavioral training could be useful for those with BID based on the fact that they can feel ownership for an artificial limb and deny it for their own limb. Further, recent data from a small case study with two BID individuals revealed that augmented reality (AR) technology could successfully reduce symptoms by engendering the illusion that the unwanted limb is missing (Turbyne et al., 2021).

Some suggest that the desire to amputate arises from a failure to adequately represent the affected limb in the relevant area of cortex. For example, McGeoch et al. (2015) recorded somatosensory evoked fields using magnetoencephalography (MEG) during tactile stimulation of the feet of BID participants. They observed a significant reduction in activity over rSPL in the patients compared to controls. A BCI approach targeting this aspect of BID may involve neurofeedback of motor evoked potentials (MEPs) in response to transcranial magnetic stimulation (TMS). This TMS-NF approach has been shown to increase (or decrease) corticospinal excitability (responsiveness) of the pathways connecting brain to muscle, depending on whether rewarding feedback was provided for large or small MEPs (Ruddy et al., 2018; Liang et al., 2020). In a BID tailored variant of this, it may be possible to incorporate feedback of MEPs from TMS over motor cortical representations of both limbs into the computer game, but bias the feedback over time to gradually reinforce engagement of the unwanted limb. This could be achieved by providing larger or more frequent rewards for large MEPs from the targeted limb. As self-regulation of MEP amplitude is achieved *via* engagement of motor imagery strategies, over time this may promote re-integration of the limb into the mental schema of body representation/body image. Reducing the discrepancy between actual and desired body representation may subsequently engender more positive affect toward the unwanted limb, albeit implicitly.

## Conclusion

As BID is a condition with both psychological and neurophysiological attributes, it is yet unclear whether interventions targeting one of these aspects in isolation can serve any benefit. Further, clarification of the relative mechanistic contribution of body ownership vs. disrupted integration of the limb into the body image will be necessary in order to accurately tailor interventions tackling these facets of the disorder. Upon reviewing the available evidence, what is known to date is that pharmacological and psychological therapies are largely ineffective, and that there are no known neurophysiological interventions that are sufficient to alleviate the suffering associated with the desire for amputation. Amputation itself is an extreme solution raising many ethical issues, and one that will not even be considered by most healthcare providers. Pretending behaviors adopted by BID patients as coping strategies raise health concerns and often have dangerous consequences. Thus, new theory driven approaches that leverage recent advances in neuroimaging and technology are greatly needed in order to improve quality of life for BID patients. Those presented here are intended to stimulate debate among those interested in working toward novel solutions, and may ultimately pave the way for a new dialog on approaches to tackle challenging neuropsychological phenomena.

## Author Contributions

SC wrote the first draft and edited the final draft. KR and SC conceptualized the idea for the manuscript. KR, SC, CS, BL, and GS worked on editing subsequent drafts of the manuscript and providing feedback. GS and BL significantly extended the sections of the manuscript. All authors reviewed the final version and were involved in discussions throughout.

## Conflict of Interest

The authors declare that the research was conducted in the absence of any commercial or financial relationships that could be construed as a potential conflict of interest.

## Publisher’s Note

All claims expressed in this article are solely those of the authors and do not necessarily represent those of their affiliated organizations, or those of the publisher, the editors and the reviewers. Any product that may be evaluated in this article, or claim that may be made by its manufacturer, is not guaranteed or endorsed by the publisher.
